# Predictors of Nonseroconversion after SARS-CoV-2 Infection

**DOI:** 10.3201/eid2709.211042

**Published:** 2021-09

**Authors:** Weimin Liu, Ronnie M. Russell, Frederic Bibollet-Ruche, Ashwin N. Skelly, Scott Sherrill-Mix, Drew A. Freeman, Regina Stoltz, Emily Lindemuth, Fang-Hua Lee, Sarah Sterrett, Katharine J. Bar, Nathaniel Erdmann, Sigrid Gouma, Scott E. Hensley, Thomas Ketas, Albert Cupo, Victor M. Cruz Portillo, John P. Moore, Paul D. Bieniasz, Theodora Hatziioannou, Greer Massey, Mary-Beth Minyard, Michael S. Saag, Randall S. Davis, George M. Shaw, William J. Britt, Sixto M. Leal, Paul Goepfert, Beatrice H. Hahn

**Affiliations:** University of Pennsylvania, Philadelphia, Pennsylvania, USA (W. Liu, R.M. Russell, F. Bibollet-Ruche, A.N. Skelly, S. Sherrill-Mix, R. Stoltz, E. Lindemuth, F.-H. Lee, K.J. Bar, S. Gouma, S.E. Hensley, G.M. Shaw, B.H. Hahn);; The University of Alabama at Birmingham, Birmingham, Alabama, USA (D.A. Freeman, S. Sterrett, N. Erdmann, M.S. Saag, R.S. Davis, W.J. Britt, S.M. Leal Jr., P. Goepfert);; Weill Cornell Medicine, New York, New York, USA (T. Ketas, A. Cupo, V.M. Cruz Portillo, J.P. Moore);; Howard Hughes Medical Institute, New York (P.D. Bieniasz);; The Rockefeller University, New York (P.D. Bieniasz, T. Hatziioannou);; Assurance Scientific, Vestavia, Alabama, USA (G. Massey, M.-B. Minyard)

**Keywords:** COVID-19, coronavirus disease, SARS-CoV-2, severe acute respiratory syndrome coronavirus 2, viruses, respiratory infections, zoonoses, nonseroconversion, RT-PCR, cycle threshold, nasopharyngeal viral loads, humoral response, serological nonresponders

## Abstract

Not all persons recovering from severe acute respiratory syndrome coronavirus 2 (SARS-CoV-2) infection develop SARS-CoV-2–specific antibodies. We show that nonseroconversion is associated with younger age and higher reverse transcription PCR cycle threshold values and identify SARS-CoV-2 viral loads in the nasopharynx as a major correlate of the systemic antibody response.

Coronavirus disease (COVID-19) is typically diagnosed by reverse transcription PCR (RT-PCR) amplification of severe acute respiratory syndrome coronavirus 2 (SARS-CoV-2) RNA from nasopharyngeal fluids (*1*). RT-PCR yields cycle threshold (C_t_) values that are inversely correlated with viral loads (*2*) and thus provide an estimate of the number of SARS-CoV-2 RNA copies in the sample. Serologic assays complement COVID-19 diagnosis by documenting past infections. In most persons, binding and neutralizing antibodies develop within 1–3 weeks after onset of symptoms (*3*), and titers correlate with disease severity (*4*).

Initial serosurveys identified antibodies in nearly 100% of persons with RT-PCR–confirmed SARS-CoV-2 infection (*5*). However, more recent studies have shown that seroconversion rates are surprisingly variable (*6*–*10*). For example, a multicenter study from Israel reported that 5% of participants remained seronegative despite a positive test result on a nasal swab specimen (*6*). In contrast, a seroprevalence study from New York found that 20% of persons with a positive RT-PCR test result did not seroconvert (*8*). Another study from Germany reported that 85% of confirmed infected COVID-19 contacts failed to develop antibodies (*9*). To examine the reasons for these differences, we investigated the relationship between seroconversion and demographic, clinical, and laboratory data in a convenience sample of convalescent persons recruited at the University of Alabama at Birmingham (Birmingham, Alabama, USA) in 2020.

## The Study

We studied 72 persons, all of whom had a previous positive RT-PCR test but were symptom-free for >3 weeks before blood was collected for testing (Table). Only 2 persons (3%) reported no symptoms, whereas 13 (18%) persons reported mild disease, 48 (67%) reported moderate disease, and 9 (12%) reported severe disease (Appendix Table 1).

**Table:** Demographic, clinical, and laboratory characteristics of serologic responders and nonresponders after SARS-CoV-2 infection*

Characteristic	SARS-CoV-2 antibody positive, n = 46	SARS-CoV-2 antibody negative, n = 26	p value†
Age, y, median (IQR)	49 (37–63)	35 (30–46)	0.03
Sex			0.17
M	30 (65)	10 (38)	
F	16 (35)	16 (62)	
Race/ethnicity			1.00
White	28 (61)	20 (77)	
Black	7 (15)	3 (12)	
Asian	7 (15)	3 (12)	
Latinx	4 (9)	0	
RT-PCR of nasal swabs			
DFOS, d, median (IQR)	5 (3–11)	5 (4–8)	0.95
C_t_ value, median (IQR)‡	24.5 (22–27)	36 (34–77)	<0.00001
Symptoms§	45 (98)	25 (96)	0.21
Severity 0	1 (2)	1 (4)	
Severity 1	5 (11)	8 (31)	
Severity 2	33 (72)	15 (58)	
Severity 3	7 (15)	2 (8)	
Hospitalization	6 (13)	2 (8)	1.00
Serologic analyses			
DFOS of T1, d, median (IQR)	34 (26–46)	33 (22–43)	0.74
Binding antibodies (positive¶)			
Spike protein (IgG)#	46 (100)	0	
Spike protein (IgA)#	43 (93)	0	
RBD (IgG)**	44 (96)	0	
RBD (IgM)**	38 (83)	0	
Nucleocapsid protein (IgG)††	43 (93)	0	
Neutralizing antibodies (positive¶)	45 (98)	0	

*Participants were a convenience sample recruited at the University of Alabama at Birmingham (Birmingham, AL, USA) during March–May 2020. Values are no. (%) unless otherwise indicated. C_t_, cycle threshold; DFOS, days following onset of symptoms; IQR, interquartile range; RBD, receptor binding domain; RT-PCR, reverse transcription PCR; SARS-CoV-2, severe acute respiratory syndrome coronavirus 2; T1, time of first serologic test. †Calculated using a likelihood ratio test for a logistic regression predicting seropositivity for the category indicated after Bonferroni correction for multiple comparisons, except for RT-PCR and serologic DFOS, for which p-values were calculated using a Welch’s 2-sample t-test. ‡C_t_ values were only available for a subset of seropositive (n = 34) and seronegative (n = 25) persons Appendix Table 1. §Symptom severity was self-reported, with 0 indicating no symptoms, 1 indicating mild symptoms with little impact on daily activities, 2 indicating moderate symptoms with noticeable impact on daily activities, and 3 indicating severe symptoms with a substantial reduction in quality of life (Appendix Table 1). ¶Above assay detection limits (Appendix Table 2 details midpoint and endpoint titers). #ELISA detection of IgG and IgA binding antibodies to a prefusion stabilized Wuhan-Hu-1 spike protein. **ELISA detection of IgM and IgG binding antibodies to RBD of the Wuhan-Hu-1 spike protein. ††Detection of IgG binding antibodies to the nucleocapsid protein by the Abbott Architect assay.

We tested plasma samples (n = 144) collected at enrollment and follow-up visits for antibodies to the spike protein by using a validated ELISA (Appendix). Only 46 of the 72 participants had detectable IgG responses, IgA responses, or both (Table); reciprocal endpoint titers ranged from 182 to >312,500 (Appendix Table 2). Analysis of the same samples for receptor-binding domain (RBD) and nucleocapsid (N) antibodies yielded very similar results (Appendix Figure 1). All persons with spike protein antibodies also had detectable RBD (IgG, IgM, or both) or N (IgG) protein responses, except for 1 participant whose spike protein endpoint titers were very low (Appendix Table 2). In contrast, 26 participants remained seronegative, despite the testing of up to 3 samples per person for IgA, IgM, and IgG against multiple antigens as well as neutralizing antibodies. Thus, 36% of our cohort represented serologic nonresponders.

**Figure 1:**
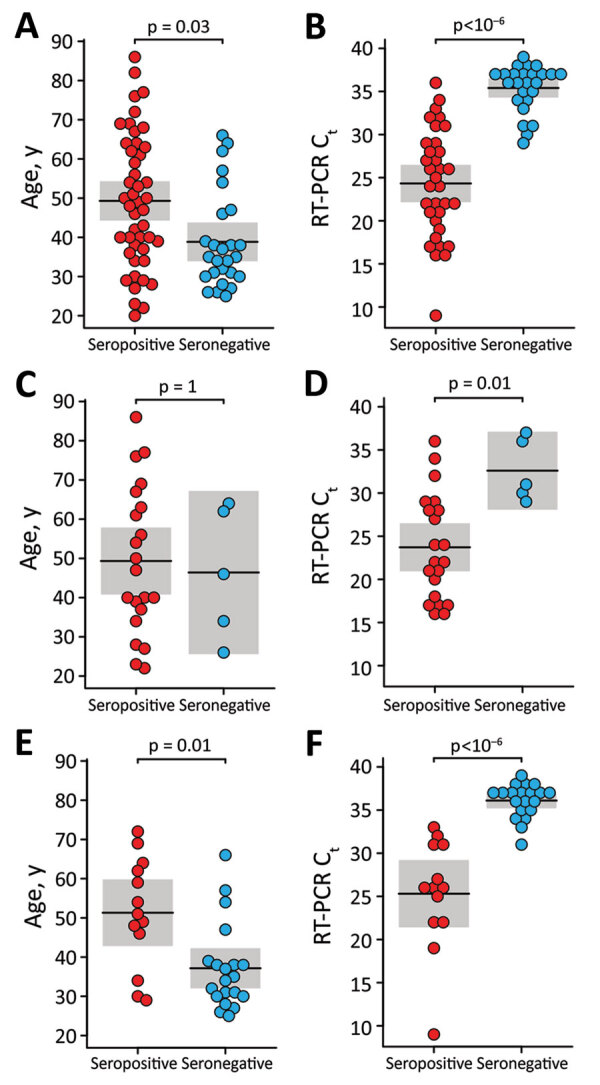
Relationship of age and nasopharyngeal viral loads with SARS-CoV-2 serostatus among convalescent persons after SARS-CoV-2 infection. Participants were a convenience sample of convalescent SARS-CoV-2–infected persons recruited at the University of Alabama at Birmingham, Birmingham, Alabama, USA, 2020. Age (panels A, C, and E) and RT-PCR C_t_ values (panels B, D, and F) are plotted for seropositive (red) and seronegative (blue) persons. Panels show comparisons of persons tested at all sites (panels A, B), the Assurance Scientific Laboratories site (panels B, C), and the University of Alabama at Birmingham Fungal Reference Laboratory and Children’s of Alabama Diagnostic Virology Laboratory sites (panels E, F). The mean (horizontal line) and corresponding 95% CI (shading) are shown; p-values indicate the results of a likelihood ratio test after Bonferroni correction for multiple comparisons. C_t_, cycle threshold; RT-PCR, reverse transcription PCR; SARS-CoV-2, severe acute respiratory syndrome coronavirus 2.

**Figure 2:**
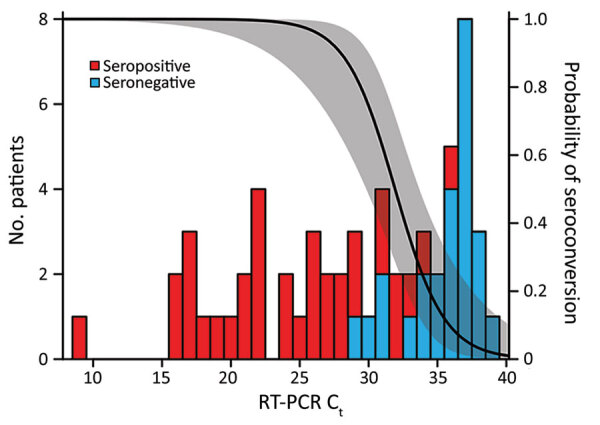
Decreasing probability of SARS-CoV-2 seroconversion with increasing RT-PCR C_t_ values among persons recovered from SARS-CoV-2 infection. Participants were a convenience sample of convalescent SARS-CoV-2–infected persons recruited at the University of Alabama at Birmingham, Birmingham, Alabama, USA, 2020. The number of serologic responders (red bars) and nonresponders (blue bars) is shown for varying RT-PCR C_t_ values. A logistic regression was used to estimate the probability of seroconversion for a given C_t_ (line) and its 95% CI (shaded). C_t_, cycle threshold; RT-PCR, reverse transcription PCR; SARS-CoV-2, severe acute respiratory syndrome coronavirus 2.

To investigate potential reasons for the lack of seroconversion, we examined available demographic, clinical, and laboratory data. Comparing race/ethnicity, sex, and symptom severity, we failed to find a significant association with serostatus (Table), although we did observe a trend for increasing antibody positivity with increasing symptom severity (Appendix Figure 2). We also found no significant differences in seroconversion between patients reporting or not reporting various symptoms, including symptoms characteristic of COVID-19 (Appendix Figure 3). However, seronegative persons were on average 10 (95% CI 3–17) years younger than seropositive persons (Figure 1, panel A) and exhibited RT-PCR C_t_ values that were 11 (95% CI 8–14) cycles higher (Figure 1, panel B). Moreover, logistic regression showed a precipitous decline in the probability of seroconversion at higher C_t_ values (Figure 2). For example, a C_t_ of 35 predicted only a 15% (95% CI 5%–37%) probability of seroconversion, which decreased further with increasing C_t_ values. Thus, low nasopharyngeal viral loads seem insufficient to elicit a systemic antibody response.

For control, we plotted C_t_ values of serologic responders and nonresponders against the times of RT-PCR and antibody testing relative to symptom onset (Appendix Figure 4). In both cases, the distributions of sampling times were similar for the 2 groups, thus excluding the possibility that seronegative persons had higher C_t_ values because they were tested too late or that they lacked antibodies because they were tested too early. We also examined remnants of purified RNA used for the initial diagnosis for the presence of SARS-CoV-2 sequences. By analyzing 12 available samples (Appendix Table 1), we were able to amplify full-length intact spike genes from 4 specimens, including 2 from seronegative persons with high C_t_ values (Appendix Figure 5).

Finally, we asked whether the relationship between seroconversion, age and C_t_ values was dependent on the diagnostic laboratory. We found that 2 sites with highly sensitive RT-PCR tests (University of Alabama at Birmingham Fungal Reference Laboratory and Children’s of Alabama Diagnostic Virology Laboratory in Birmingham) were 6 (95% CI 2–30) times more likely to identify serologic nonresponders than a third site with a less sensitive test (Assurance Scientific Laboratories in Birmingham) (Appendix Methods). However, this difference did not change the relationship between C_t_ values and seroconversion because seronegative persons had higher C_t_ values than seropositive persons regardless of the test site (Figure 1, panels D, F). In contrast, we observed little association between age and seroconversion at the Assurance Scientific Laboratories site (Figure 1, panel C), and the difference observed at the other sites was largely driven by young persons who also had high C_t_ values (Figure 1, panel E). Thus, nasopharyngeal viral loads represent a major correlate of the systemic antibody response, whereas age seems to have only a minor effect.

## Conclusions

In summary, we show that patients with low SARS-CoV-2 viral loads in their respiratory tract are less likely to mount a systemic antibody response. Although we cannot formally exclude false-positive RT-PCR results in some participants, PCR contamination is highly unlikely as an explanation for our findings (Appendix). We also show that clinical illness does not guarantee seroconversion and that laboratories with highly sensitive RT-PCR assays are more likely to detect serologic nonresponders. These results provide an explanation for the puzzling variability of seroconversion in different cohorts.

The fact that a considerable fraction of RT-PCR positive persons fail to seroconvert has practical implications. Such persons remain undetected in seroprevalence studies, including in vaccine studies that assess protection from asymptomatic infection by measuring antibodies to antigens not included in the vaccine. Seroconverters and nonseroconverters will probably also respond differently to vaccination. Recent studies revealed that seropositive persons have a heightened antibody response after the first, but not the second, dose of an mRNA vaccine, suggesting that a single dose is sufficient (*11**–**13*; Samanovic et al., unpub. data, https://doi.org/10.1101/2021.02.07.21251311). Serologic nonresponders might not exhibit a similarly heightened anamnestic response, but resemble SARS-CoV-2 naive persons, as was observed for 1 previously infected vaccinee who never seroconverted (*14*). Finally, RT-PCR positive persons who experienced COVID-19 symptoms might be less inclined to seek vaccination, believing they are protected, but our results caution against this assumption.

Appendix Additional information about predictors of nonseroconversion after SARS-CoV-2 infection.
