# Supplementary material for: Predictors of Nonseroconversion after SARS-CoV-2 Infection
Source: Emerg Infect Dis. 2021 Sep;27(9):2454–8. doi: 10.3201/eid2709.211042 (PMC8386781; doi:10.3201/eid2709.211042)
Supplement: Appendix — Additional information about predictors of nonseroconversion after SARS-CoV-2 infection. [file 21-1042-Techapp-s1.pdf]

Article DOI: https://doi.org/10.3201/eid2709.211024

Predictors of Nonseroconversion after SARS-CoV-2 Infection

Appendix

Methods

Ethics and cohort characteristics

The University of Alabama at Birmingham (UAB) COVID-19 convalescent cohort was established in March 2020 at the 1917 Clinic and recruited 72 persons by May 2020. Many participants were UAB employees who were made aware of this cohort after being informed that they had tested positive for COVID-19. Part of the initial phone call included information about the study, which was designed to evaluate immune responses following SARS-CoV-2 infection. Participants also heard about the study by word of mouth from other patients or health care providers. All persons were given information where they could schedule an appointment to provide informed consent and enroll into the study, but they were not asked whether they intended to participate. Thus, the fraction of patients who decided not to enroll is unknown. A potential sampling bias includes a predominance of health care professionals interested in the potency of their antiviral immune responses and persons motivated to advance scientific knowledge about SARS-CoV-2 infection and disease.

All participants were enrolled after obtaining written informed consent and approval from the Institutional Review Board (IRB-160125005). Participants had a median age of 40 years (range 20–86 years), were 56% male and 44% female, and had diverse racial/ethnic backgrounds (67% Caucasian, 14% African American, 14% Asian, 5% Latinx). Symptom severity was self-reported, with 0 indicating no symptoms, 1 indicating mild symptoms with little impact on daily activities, 2 indicating moderate symptoms with noticeable impact on daily activities, and 3 indicating severe symptoms with a significant reduction in quality of life (Appendix Table 1). Data on hospital admission and stay were obtained from electronic medical records. Eight of the

nine persons with severe symptoms were hospitalized (Appendix Table 1). Blood samples were collected longitudinally under the appropriate IRB guidelines.

RT-PCR

All study participants were confirmed to be SARS-CoV-2 infected as determined by RT-PCR analysis of nasopharyngeal swabs (Appendix Table 1). Of the 72 convalescent persons, 13 were diagnosed at clinical laboratories that reported only positive or negative results. However, the remaining 59 participants were tested at one of three laboratories, which provided quantitative C_t values and were Clinical Laboratory Improvement Amendments (CLIA) certified. These included the UAB Fungal Reference Laboratory (FRL), the Children's of Alabama Diagnostic Virology Laboratory (CoA), and the Assurance Scientific Laboratories (ASL). RNA was extracted from transport medium using the Omega Viral RNA manual extraction kit (FRL), the Roche MagnaPure (CoA), the Abnova Total Nucleic Acid Purification Kit (ASL) or the Zymo Research Quick-DNA/RNA Viral MagBead Kit (ASL) and subjected to RT-PCR using the N1 primer set from the Centers for Disease Control (CDC) 2019-nCoV RT-PCR Diagnostic Panel (Integrated DNA Technologies) and human RNase P primers for control. RT-PCR reactions were run using the ThermoFisher TaqMan Fast Virus 1-Step Master Mix (FRL, CoA) or the ThermoFisher TaqPath 1-Step RT-qPCR Master Mix (ASL). RT-PCR was performed on the ThermoFisher QuantStudio 5 (FRL), the ThermoFisher QuantStudio 6 (CoA), or the BioRad CFX384 (ASL). All three laboratories determined the limits of detection (LoD) of their RT-PCR tests by using an FDA reference panel for SARS-CoV-2 nucleic acid-based amplification tests (NAAT). These LoD values were 180 RNA NAAT detectable units (NDU) per ml for FRL, 360 NDU/ml for CoA, and 5,400 NDU/ml for ASL. In addition, all three laboratories included multiple controls (no extraction control, no template control, positive template control) in each RT-PCR reaction to minimize false positive and false-negative results as described in their Emergency Use Authorization (*1*,*2*).

SARS-CoV-2 S protein ELISA

IgG and IgA binding antibodies to the viral spike (S) protein were detected by enzyme-linked immunosorbent assay (ELISA) using a recombinantly expressed, pre-fusion stabilized (Wuhan-Hu-1) S-protein as previously described (*3*,*4*). Briefly, Costar high binding flat-bottom 96-well plates were coated with 300 ng of a recombinantly expressed, pre-fusion stabilized (S-2P) Wuhan-Hu-1 (residues 1–1138) S-protein (plasmid kindly provided by Philip Brouwer and

Rogier W. Sanders, Department of Medical Microbiology, University of Amsterdam, Amsterdam, The Netherlands) in PBS overnight at 4°C and then blocked with blocking buffer (5% non-fat milk powder in PBS + 0.05% Tween 20) for 1 h at 37°C. Plasma samples were heat-inactivated at 56°C for 1 hour, 5-fold serially diluted in blocking buffer and then added to the plates for 1 h at 37°C. After five washes with PBS-T (PBS + 0.1% Tween 20), plates were incubated for 1 h at 37°C with horse radish peroxidase (HRP)-conjugated goat-anti-human IgA and IgG detection antibodies diluted 1:5,000 in blocking buffer. After five additional washes, 3,3′,5,5′-tetramethylbenzidine (TMB) substrate was added for color development for 10 min before the reaction was stopped with an equal volume of 1N H_2SO_4. Absorbance was read at 450 nm using a Synergy 4 spectrophotometer. The average OD_{450} value from three background control wells (no plasma) was subtracted from the S-protein coated wells. In addition, the average OD_{450} value (plus two standard deviations) of 28 pre-pandemic sera was subtracted from each plasma dilution. Midpoint (EC_{50}) and endpoint titers were determined as described (*5*). Briefly, midpoint (EC_{50}) titers were calculated by a nonlinear-regression fit of a 4-parameter sigmoid function to the corrected OD_{450} values and the logarithmic dilution factors (the lower plateau was set to 0; GraphPad Prism software). End-point titers were read from the fitted curve at a corrected OD_{450} cutoff of 0.1.

SARS-CoV-2 receptor binding domain ELISA

IgG and IgM binding antibodies to the receptor binding domain (RBD) of the Wuhan-Hu-1 spike protein were detected by ELISA as described (*6*). Briefly, SARS-CoV-2 RBD protein (spike residues 419–541) was expressed in 293F cells (plasmid kindly provided by Florian Krammer, Icahn School of Medicine at Mount Sinai, New York, USA) and purified. ELISA plates were coated overnight at 4°C with 100 ng of recombinant RBD diluted in PBS, washed 3 times with PBS-T, and blocked for 1 hour with PBS-T supplemented with 3% non-fat milk powder. Plasma samples were heat-inactivated at 56°C for 1 hour, 2-fold serially diluted and then added to the plates for 2 hours at room temperature. After 3 washes with PBS-T, horseradish peroxidase (HRP) labeled goat anti-human IgG or goat anti-human IgM detection antibodies were incubated for 1h at room temperature. Plates were washed 3 times with PBS-T and TMB substrate was added for color development for 5 min before the reaction was stopped with H_2SO_4. Absorbance was read at 450 nm using a SpectraMax 190 microplate reader. Background OD_{450} values from plates coated with PBS were subtracted from OD_{450} values from

RBD coated plates. A dilution series of the IgG monoclonal antibody CR3022, which binds the SARS-CoV-2 spike protein, was included on all plates as a control for inter assay variability. Serum antibody concentrations were reported as arbitrary units defined as the relative ratio of sample and control antibody (CR3022) OD_{450} values of at 4 ng/ml.

SARS-CoV-2 nucleocapsid ELISA

Antibodies to the SARS-CoV-2 nucleocapsid protein were determined using the Abbott Architect, a commercially available chemiluminescent microparticle immunoassay (CMIA) (*7*). The quantity of detected IgG is reported as a signal-to-cutoff index, with values over 1.4 considered positive for N protein antibodies.

SARS-CoV-2 pseudovirus neutralization assay

Plasma samples were tested for SARS-CoV-2 neutralizing antibodies as previously described (*8*) using an HIV-1 based pseudovirus assay. Briefly, the SARS-CoV-2 Spike (D614G variant, with a 19 aa cytoplasmic tail deletion) was pseudotyped onto an HIV-1 nanoluciferase encoding reporter backbone by co-transfection in HEK 293T cells. Pseudovirus was incubated with 5-fold serial dilutions of patient plasma and then used to infect 1.5×10^4 293T clone 22 cells expressing ACE2. Two days post-infection, cells were washed with PBS, lysed, and nanoluciferase activity was determined according to manufacturer’s instructions (Nano-Glo® Luciferase Assay System). Luciferase activity in wells with virus and no patient plasma were set to 100%, and the dilution of plasma at which luminescence was reduced to 50% (Inhibitory Dose 50; ID_{50}) was calculated as an average of two technical duplicates.

Amplification of full-length spike sequences from nasal swabs

Left-over viral transport medium or remnant extracted RNA used for the initial SARS-CoV-2 diagnosis were obtained from the clinical laboratories. Only 12 such samples could be identified, four of which were from seropositive and eight from seronegative persons. cDNA was generated using primer WHCV-S-R1 (5′-CAAAGTTACAGTTCCAATTGTGAAG-3′) and Superscript III reverse transcription. The full-length spike gene was amplified by nested PCR using High Fidelity Taq polymerase and primers WHCV-S-F1 (5′-AGTAAAGGTAGACTTATAATTAGAGAA-3′) and WHCV-S-R1 in the first round, and WHCV-S-F2 (5′-TTCTAGTGATGTTCTTGTTAACAAC-3′) and WHCV-S-R2 (5′-TTCTCATAAACAAATCCATAAGTTCG-3′) in the second round, respectively. Amplification

conditions included an initial denaturation step of 2 minutes at 94°C, followed by 37 cycles (first round) or 40 cycles (second round) of denaturation (94°C, 18 sec), annealing (52°C, 30 sec or 54°C, 30 sec), and elongation (68°C, 4 min 20 sec), followed by a final elongation step of 5 min at 68°C. Amplicons were MiSeq sequenced and analyzed using Geneious 11.0.4. All contained the D614G spike mutation (Appendix Figure 5), consistent with the geographic distribution of this variant at the time of sampling (*9*). Sequences were deposited in GenBank under accession codes MZ027643 to MZ027646.

Statistical analyses

Logistic regression was used to individually examine the association of seroconversion status with race/ethnicity, gender, symptom severity, hospitalization, age, RT-PCR C_t values and the presence of various symptoms. Significance was assessed using a likelihood ratio test and corrected for multiple comparisons using Bonferroni correction (n = 6 for race/ethnicity, gender, symptom severity, hospitalization, age and C_t values; n = 4 for age and C_t values by site; n = 16 for symptomatology). The combined effects of age and RT-PCR C_t were assessed using a likelihood ratio test after multivariate logistic regression analysis. Data were analyzed using R v4.0.5 (*10*).

Serostatus and symptoms

The strength of the humoral SARS-CoV-2 immune response is known to correlate with disease severity (*11–13*), which is consistent with recent findings that asymptomatic persons seroconvert at a lower rate (*14,15*). In our cohort, the great majority of participants were symptomatic, including 25 of 26 serologic non-responders, all but one reported one or more case definition symptoms of COVID-19 (Appendix Table 1), such as cough, shortness of breath and/or sudden onset of loss of smell or taste (*16*). Thus, the serologic non-responder phenotype is not limited to asymptomatic persons, but is also found among persons who recovered from mild and moderate COVID-19.

Serostatus and age

Across our cohort, persons who failed to seroconvert were younger than their antibody positive counterparts. This observation is consistent with the findings of a Swiss study, which reported that titers of mucosal IgA were inversely correlated with age and could be present even in the absence of serum IgA and IgG, suggesting that younger persons are more likely to mount a

mucosal antibody response (*17*). Younger persons may also develop more vigorous innate responses and counteract new infections more effectively since they have larger repertoires of naïve immune cells (*18*,*19*). Thus, RT-PCR positive persons who fail to seroconvert may control SARS-CoV-2 replication at the portal of entry, limiting the accumulation of infectious virus and viral antigen. However, it is also possible that in at least some cases high C_t values are indicative of small amounts of SARS-CoV-2 nucleic acids that do not represent replication competent virus.

Quality control

In this study, we failed to detect SARS-CoV-2 specific antibodies in the plasma of a surprisingly large proportion (36%) of 72 COVID-19 convalescent persons. This was not due to false negative test results since we used multiple serologic approaches, including a widely used commercial assay (*7*). This was also not due to insufficient sampling, since testing of multiple samples against different antigens and antibody isotypes yielded identical results (Appendix Table 2). While we cannot formally exclude false positive RT-PCR results for some participants, PCR contamination is highly unlikely as an explanation for our findings for several reasons. First, serologic non-responders were identified by three different diagnostic laboratories (Appendix Table 1), all of which employed stringent quality control measures to guard against false-positive results. Second, we were able to independently amplify SARS-CoV-2 sequences from a subset of the original nasal swab material. Analyzing 4 samples from seropositive and 8 samples from seronegative persons (Appendix Table 1), we amplified full-length spike genes with intact open reading frames from four specimens, including two from seronegative persons (Appendix Figure 5). Finally, RT-PCR positive seronegative persons have also been identified by several other groups (15, 20, 21; Dash et al., unpub. data, https://doi.org/10.1101/2020.11.13.20229716), all of which showed that nasal swabs from these persons had significantly higher C_t values than their seropositive counterparts.

References

1. US Food and Drug Administration. Emergency Use Authorization (EUA) summary. Assurance SARS-COV-2 panel (Assurance Scientific Laboratories). For in vitro diagnostic use Rx only. For use under Emergency Use Authorization (EUA) only [cited 2021 Jun 24]. https://www.fda.gov/media/138154/download

2. US Food and Drug Administration. Accelerated Emergency Use Authorization (EUA) summary. FRL SARS-CoV-2 Test (UAB Fungal Reference Lab). For in vitro diagnostic use Rx only. For use under Emergency Use Authorization (EUA) only [cited 2021 Jun 24]. https://www.fda.gov/media/139437/download

3. Brouwer PJM, Caniels TG, van der Straten K, Snitselaar JL, Aldon Y, Bangaru S, et al. Potent neutralizing antibodies from COVID-19 patients define multiple targets of vulnerability. Science. 2020;369:643–50. PubMed https://doi.org/10.1126/science.abc5902

4. Ketas TJ, Chaturbhuj D, Portillo VMC, Francomano E, Golden E, Chandrasekhar S, et al. Antibody responses to SARS-CoV-2 mRNA vaccines are detectable in saliva. Pathog Immun. 2021;6:116–34. PubMed https://doi.org/10.20411/pai.v6i1.441

5. Honjo K, Russell RM, Li R, Liu W, Stoltz R, Tabengwa EM, et al. Convalescent plasma-mediated resolution of COVID-19 in a patient with humoral immunodeficiency. Cell Rep Med. 2020;2:100164. PubMed https://doi.org/10.1016/j.xcrm.2020.100164

6. Flannery DD, Gouma S, Dhudasia MB, Mukhopadhyay S, Pfeifer MR, Woodford EC, et al. SARS-CoV-2 seroprevalence among parturient women in Philadelphia. Sci Immunol. 2020;5:eabd5709. PubMed https://doi.org/10.1126/sciimmunol.abd5709

7. Chew KL, Tan SS, Saw S, Pajarillaga A, Zaine S, Khoo C, et al. Clinical evaluation of serological IgG antibody response on the Abbott Architect for established SARS-CoV-2 infection. Clin Microbiol Infect. 2020;26:1256.e9–11. PubMed https://doi.org/10.1016/j.cmi.2020.05.036

8. Schmidt F, Weisblum Y, Muecksch F, Hoffmann HH, Michailidis E, Lorenzi JCC, et al. Measuring SARS-CoV-2 neutralizing antibody activity using pseudotyped and chimeric viruses. J Exp Med. 2020;217:e20201181. PubMed https://doi.org/10.1084/jem.20201181

9. Korber B, Fischer WM, Gnanakaran S, Yoon H, Theiler J, Abfalterer W, et al.; Sheffield COVID-19 Genomics Group. Tracking changes in SARS-CoV-2 spike: evidence that D614G increases infectivity of the COVID-19 virus. Cell. 2020;182:812–827.e19. PubMed https://doi.org/10.1016/j.cell.2020.06.043

10. R Core Team. R: a language and environment for statistical computing. 2017 [cited 2021 Jun 24]. https://www.R-project.org

11. Gaebler C, Wang Z, Lorenzi JCC, Muecksch F, Finkin S, Tokuyama M, et al. Evolution of antibody immunity to SARS-CoV-2. Nature. 2021;591:639–44. PubMed https://doi.org/10.1038/s41586-021-03207-w

12. Wang P, Liu L, Nair MS, Yin MT, Luo Y, Wang Q, et al. SARS-CoV-2 neutralizing antibody responses are more robust in patients with severe disease. Emerg Microbes Infect. 2020;9:2091–3. PubMed https://doi.org/10.1080/22221751.2020.1823890

13. Rijkers G, Murk JL, Wintermans B, van Looy B, van den Berge M, Veenemans J, et al. Differences in antibody kinetics and functionality between severe and mild severe acute respiratory syndrome coronavirus 2 infections. J Infect Dis. 2020;222:1265–9. PubMed https://doi.org/10.1093/infdis/jiaa463

14. Milani GP, Dioni L, Favero C, Cantone L, Macchi C, Delbue S, et al.; UNICORN Consortium. Serological follow-up of SARS-CoV-2 asymptomatic subjects. Sci Rep. 2020;10:20048. PubMed https://doi.org/10.1038/s41598-020-77125-8

15. Wellinghausen N, Plonné D, Voss M, Ivanova R, Frodl R, Deininger S. SARS-CoV-2-IgG response is different in COVID-19 outpatients and asymptomatic contact persons. J Clin Virol. 2020;130:104542. PubMed https://doi.org/10.1016/j.jcv.2020.104542

16. Centers for Disease Control and Prevention. National Notifiable Diseases Surveillance System: coronavirus disease 2019 (COVID-19) 2020 interim case definition, approved August 5, 2020 [cited 2021 Jun 24]. https://ndc.services.cdc.gov/case-definitions/coronavirus-disease-2019-2020-08-05

17. Cervia C, Nilsson J, Zurbuchen Y, Valaperti A, Schreiner J, Wolfensberger A, et al. Systemic and mucosal antibody responses specific to SARS-CoV-2 during mild versus severe COVID-19. J Allergy Clin Immunol. 2021;147:545–557.e9. PubMed https://doi.org/10.1016/j.jaci.2020.10.040

18. Brodin P, Davis MM. Human immune system variation. Nat Rev Immunol. 2017;17:21–9. PubMed https://doi.org/10.1038/nri.2016.125

19. Bajaj V, Gadi N, Spihlman AP, Wu SC, Choi CH, Moulton VR. Aging, immunity, and COVID-19: how age influences the host immune response to coronavirus infections? Front Physiol. 2021;11:571416. PubMed https://doi.org/10.3389/fphys.2020.571416

20. Masiá M, Telenti G, Fernández M, García JA, Agulló V, Padilla S, et al. SARS-CoV-2 seroconversion and viral clearance in patients hospitalized with COVID-19: viral load predicts antibody response. Open Forum Infect Dis. 2021 Jan 5 [Epub ahead of print]. PubMed https://doi.org/10.1093/ofid/ofab005

21. Thiruvengadam R, Chattopadhyay S, Mehdi F, Desiraju BK, Chaudhuri S, Singh S, et al.; DBT India Consortium for COVID 19 Research. Longitudinal serology of SARS-CoV-2-infected individuals in India: a prospective cohort study. Am J Trop Med Hyg. 2021 May 18 [Epub ahead of print]. PubMed

Appendix Table 1. Demographic, clinical and laboratory characteristics of 72 persons who recovered from SARS-CoV-2 infection*

			Race/	Nucleic acid test					Symptom		Antibody tests	
ID	Age	Sex	ethnicity†	Date	DFOS	C_t (Lab)‡	Spike PCR	Symptoms§	severity¶	Hospitalization#	DFOS	Seroconversion
CR0001	57	F	CC	3/23/20	32	38 (FRL)	Neg	AA, CO, FA	1	No	41, 60, 119	No
CR0003	22	M	CC	3/20/20	11	24 (ASL)	ND	AA, CT, DI, DY, MY	1	No	23, 42, 99	Yes
CR0004	54	M	CC	3/23/20	7	38 (FRL)	ND	CO, CT, DY, FA, MY, PD, ST	2	No	16, 35, 86	No
CR0005	34	F	AA	3/14/20	4	22 (ASL)	ND	CH, CO, FE, HE, MY, NC, NV, ST	2	No	24, 41, 104	Yes
CR0006	26	M	CC	3/18/20	4	30 (ASL)	Neg	AA, CH, CO, CT, DY, FA, FE, HE, MY, NC, NS, NV, PD, RH, ST	2	No	20, 39, 103	No
CR0007	28	M	CC	3/19/20	2	32 (ASL)	ND	CH, FA, FE, HE, MY, NC	2	No	20, 38	Yes
CR0008	38	F	CC	3/18/20	10	34 (FRL)	Neg	AA, CO, CT, DY, FA, MY, NC	1	No	29	No
CR0010	42	F	CC	3/16/20	3	NA	ND	AA, CH, CO, CT, DI, DY, FA, FE, HE, MY, NS, NV, PD	2	No	3, 39, 101	Yes
CR0011	64	M	CC	3/16/20	3	26 (CoA)	ND	AA, CH, CO, FA, FE, HE, MY, NC, PD, RH	2	No	25, 39, 48	Yes
CR0012	40	M	CC	3/18/20	2	27 (ASL)	ND	AA, CH, CO, CT, DY, FA, HE, MY, NC, NS, PD, RH	2	No	22, 35	Yes
CR0014	69	M	CC	3/19/20	−7	20 (ASL)	ND	CO, DI, DY, FA, FE, HE, NS	2	No	14, 28	Yes
CR0016	31	F	CC	3/30/20	4	37 (FRL)	Neg	AA, FA, HE, MY	2	No	13, 27, 95	No
CR0017	46	F	CC	3/27/20	9	33 (FRL)	ND	AA, CO, NC	1	No	21	Yes
CR0019	61	M	CC	3/18/20	2	29 (ASL)	ND	CH, CO, DI, FA, FE, HE, MY, NS, NV, PD	2	No	23, 36, 93	Yes
CR0020	39	F	CC	3/24/20	4	37 (FRL)	Pos	CH, CT, DI, DY, FA, FE, HE, MY, NS	2	No	19, 34	No
CR0021	76	M	CC	3/19/20	7	17 (ASL)	ND	CO, FA, MY, RH	2	No	28, 40, 96	Yes
CR0022	32	M	CC	3/21/20	14	37 (FRL)	Pos	AA, CO, DI, DY, FA, FE, MY, NS, NV, PD, RH	3	Yes	33, 46	No
CR0023	46	F	CC	3/17/20	4	29 (ASL)	ND	CH, CO, CT, DI, DY, FA, HE, MY, NS, PD, RH	1	No	27, 47	No
CR0024	47	M	CC	3/24/20	5	37 (FRL)	Neg	CH, CO, CT, DY, FA, FE, HE, MY, PD	1	No	22, 36	No
CR0025	50	M	CC	3/19/20	12	24 (ASL)	ND	AA, CH, CO, CT, DY, FA, FE, HE, MY	2	No	34, 47, 103	Yes
CR0026	30	F	AS	3/23/20	5	9 (FRL)	ND	AA, CO, FA, FE, HE, MY	2	No	26, 34	Yes
CR0027	63	M	AS	3/18/20	2	16 (ASL)	ND	AA, CH, CO, DY, FA, FE, HE, MY, PD	2	No	28, 99	Yes
CR0028	26	F	CC	3/30/20	6	37 (FRL)	ND	CO, DY, FE, HE, NC, RH	1	No	21	No
CR0030	29	M	CC	3/25/20	4	19 (FRL)	ND	AA, CH, CT, DI, DY, FA, FE, HE, MY, NC, NS, NV, RH, ST	2	No	24, 32	Yes
CR0032	66	F	CC	3/25/20	−3	34 (FRL)	ND	CO	2	No	20, 35	No
CR0033	27	F	CC	3/19/20	3	17 (ASL)	ND	AA, CH, CT, DI, DY, FA, FE, HE, NC, NS, PD, ST	3	Yes	30, 37	Yes
CR0035	27	M	CC	3/24/20	6	35 (FRL)	ND	AA, CO, FA, HE, MY, NC	2	No	29, 34, 92	No
CR0037	38	M	CC	3/16/20	1	NA	ND	DY, HE, MY	2	No	32, 39, 102	Yes
CR0038	56	F	AS	3/18/20	4	17 (ASL)	ND	AA, CO, CT, DY, FA, HE, MY	2	No	33, 40, 95	Yes
CR0039	67	M	AS	3/19/20	2	22 (ASL)	ND	AA, CH, CO, CT, DI, DY, FA, FE, HE, MY, NS, NV, PD, ST	2	No	30, 37	Yes
CR0042	54	M	LA	3/23/20	13	28 (ASL)	ND	CH, CO, DI, FA, FE, HE, NS, PD, RH	1	No	41, 59	Yes
CR0043	86	F	CC	3/24/20	9	21 (ASL)	ND	AA, CT, DI, FA, FE, HE, NC, ST	2	No	37, 45	Yes
CR0045	64	F	LA	3/27/20	10	NA	ND	AA, CO, CT, DI, DY, FE, MY	2	No	35, 43	Yes
CR0046	29	M	CC	3/30/20	3	NA	ND	AA, CH, CO, DY, FE, MY	3	Yes	24	Yes
CR0048	34	M	CC	3/19/20	7	22 (CoA)	ND	CO, FA, FE, HE, PD	1	No	34, 77	Yes
CR0050	40	M	CC	4/2/20	11	NA	ND	CT, DY	2	No	31, 38, 95	Yes
CR0051	64	F	CC	3/27/20	2	36 (ASL)	ND	DY, FA, FE, HE, MY, PD	2	No	29, 36	No
CR0054	49	F	LA	3/22/20	10	26 (FRL)	ND	CH, CO, CT, DI, DY, FA, FE, HE, MY, NS, NV, PD, ST	3	Yes	43, 106	Yes
CR0055	62	M	AA	4/1/20	10	31 (ASL)	ND	CO, CT, DY, FE, HE, NC, PD, RH, ST	2	No	33, 40	No
CR0057	62	M	CC	3/22/20	9	25 (FRL)	ND	CH, CO, DI, DY, FE, HE, MY, NS, NV	3	Yes (ICU)	45, 97	Yes
CR0060	51	M	AS	4/2/20	4	32 (FRL)	Pos	AA, CH, CO, DY, FA, FE, HE, MY, NS, NV, PD	3	No	29, 88	Yes
CR0061	50	F	LA	3/31/20	2	NA	ND	AA, CH, CO, CT, DI, DY, FA, FE, HE, MY, NV, PD	2	No	29, 44, 82	Yes
CR0062	25	F	CC	3/28/20	5	38 (FRL)	ND	CH, CO, CT, DY, FA, FE, HE, MY, NC, NS, NV, RH	2	No	36, 46	No
CR0064	82	M	CC	3/23/20	7	NA	ND	CH, CO, DI, FA, FE, HE, MY, NC, NS, PD, RH	2	No	43, 49	Yes
CR0066	72	M	CC	4/4/20	14	27 (FRL)	Pos	AA, CO, DI, DY, FA, HE, MY, NC, RH	3	Yes	39	Yes
CR0067	39	M	CC	3/13/20	15	29 (ASL)	ND	AA, CH, CO, CT, DY, FA, FE, MY, NS, PD	2	No	62, 81, 124	Yes
CR0068	43	M	CC	3/18/20	NA	NA	ND	No symptoms	0	No	43, 93	Yes

ID	Age	Sex	Race/ ethnicity†	Nucleic acid test Date	DFOS	C_t (Lab)‡	Spike PCR	Symptoms§	Symptom severity¶	Hospitalization#	Antibody tests DFOS	Seroconversion
CR0069	37	M	CC	3/18/20	20	34 (ASL)	ND	AA, DI, FA, NC	2	No	62	Yes
CR0070	28	F	CC	3/23/20	5	35 (FRL)	ND	CT, DY, FA, FE, HE, MY, NC, PD	2	No	42, 63	No
CR0071	54	F	AA	4/4/20	14	31 (FRL)	Neg	AA, CT, DY, FE, HE, NS, NV, PD, RH	3	Yes	40, 94	Yes
CR0072	38	F	AA	3/28/20	2	33 (FRL)	ND	CO	1	No	35, 54	No
CR0073	69	F	CC	3/17/20	5	31 (FRL)	ND	CH, CO, CT, DY, FA, HE, MY, NC, NS, NV, PD, RH	2	No	49, 92	Yes
CR0074	48	F	AA	3/31/20	12	22 (FRL)	Neg	CH, CO, DI, FA, FE, HE, MY, NS, PD	2	No	43	Yes
CR0078	40	M	AS	3/18/20	16	36 (ASL)	ND	AA, CH, DI, FA, HE, MY	2	No	60, 102	Yes
CR0079	40	M	AS	4/7/20	4	21 (ASL)	ND	AA, HE, PD	2	No	31	Yes
CR0082	30	F	AS	3/31/20	5	39 (FRL)	ND	CO, DY, FA	1	No	40, 56	No
CR0083	31	F	CC	3/28/20	6	36 (FRL)	ND	AA, CO, CT, DY, FA	1	No	44, 60	No
CR0086	68	F	CC	3/23/20	10	NA	ND	AA, CH, CO, DI, DY, FA, FE, HE, MY, NS	2	No	50, 108	Yes
CR0087	38	M	AA	3/28/20	3	36 (FRL)	ND	CO	2	No	43, 54	No
CR0089	35	M	AS	3/23/20	3	31 (FRL)	ND	DI, FA, FE, MY, NC	2	No	49, 68	No
CR0090	35	F	CC	3/13/20	17	NA	ND	CH, CO, CT, DY, FA, FE, HE, MY, NC, NS, NV, PD, RH	2	No	76, 111	No
CR0093	30	F	CC	3/20/20	9	37 (FRL)	ND	AA, CH, CO, CT, DY, FA, FE, HE, MY, NS, NV, PD, RH, ST	3	Yes	63, 83	No
CR0094	53	F	AA	4/2/20	10	NA	ND	CO, DI, DY, FA, FE, HE, MY, NS, NV, PD	2	No	52, 93	Yes
CR0095	20	F	AA	3/24/20	3	NA	ND	AA, CO, FA, FE, HE, MY, NS, PD	2	No	54	Yes
CR0098	23	M	AA	4/3/20	5	18 (ASL)	ND	AA, CT, DY, FA, FE, HE, MY, NC, RH	2	No	50	Yes
CR0099	34	M	AS	3/23/20	2	37 (FRL)	ND	CH, CO, DI, FA, HE, MY, NV	2	No	58	No
CR0100	59	M	CC	3/31/20	4	26 (FRL)	ND	AA, CO, FA, HE	2	No	53	Yes
CR0101	37	F	CC	3/23/20	6	36 (FRL)	ND	CH, CO, CT, DI, DY, FA, HE, NC, NS, PD	2	No	63	No
CR0102	77	M	CC	3/13/20	5	16 (ASL)	ND	DI, FA, NS, NV	2	No	73	Yes
CR0104	47	M	AA	4/27/20	11	28 (ASL)	ND	AA, CO, CT, DI, DY, HE, MY, NC	2	No	36	Yes
CR0105	36	M	CC	3/21/20	5	NA	ND	CT, DY, FE	1	No	72	Yes
CR0108	34	M	CC	5/4/20	NA	37 (ASL)	Neg	No symptoms	0	No	25	No

*SARS-CoV-2, severe acute respiratory syndrome-coronavirus-2; DFOS, days following onset of symptoms (not available for participants CR0068 and CR0108, who were asymptomatic). NA, not available; ND, not done.
†CC, Caucasian; AA, African American; AS, Asian; LA, Latinx.
‡C_t, cycle threshold; FRL, Fungal Reference Laboratory; ASL, Assurance Scientific Laboratories; CoA, Children's of Alabama Diagnostic Virology Laboratory.
§AA, anosmia/ageusia; CH, chills; CO, cough; CT, chest tightness; DI, diarrhea; DY, dyspnea; FA, fatigue; FE, fever >100.4°F; HE, headache; MY, myalgia; NC, nasal congestion; NS, night sweats; NV, nausea/vomiting; PD, psychataxia/dizziness; RH, rhinorrhea; ST, sore throat (also see Appendix Figure 3).
¶Symptom severity was self-reported, with 0 indicating no symptoms, 1 indicating mild symptoms with little impact on daily activities, 2 indicating moderate symptoms with noticeable impact on daily activities, and 3 indicating severe symptoms with a significant decrease in quality of life.
#One hospitalized patient was admitted to the Intensive Care Unit (ICU).

Appendix Table 2. Binding and neutralizing antibody titers in the plasma of 72 persons with confirmed SARS-CoV-2 infection*

			Binding antibodies†							
			Spike (S protein)				RBD		N	Neutralizing antibodies
			IgG		IgA		IgG	IgM	IgG	D614G‡
Sample	Date	DFOS	Endpoint titer	EC_{50}	Endpoint titer	EC_{50}	Arbitrary units	Arbitrary units	Index	ID_{50}
CR0001-1	4/1/20	41	<100	<100	<100	<100	<0.20	<0.20	0.05	<20
CR0001-2	4/20/20	60	<100	<100	<100	<100	<0.20	<0.20	0.05	<20
CR0001-3	6/18/20	119	<100	NA	<100	NA	NA	NA	NA	<20
CR0003-1	4/1/20	23	1,112	<100	559	<100	0.20	<0.20	1.30	56
CR0003-2	4/20/20	42	20,427	908	2,489	<100	2.59	<0.20	2.51	346
CR0003-3	6/16/20	99	9,285	NA	613	NA	NA	NA	NA	124
CR0004-1	4/1/20	16	<100	<100	<100	<100	<0.20	<0.20	neg	<20
CR0004-2	4/20/20	35	<100	<100	<100	<100	<0.20	<0.20	0.02	<20
CR0004-3	6/10/20	86	<100	NA	<100	NA	NA	NA	NA	<20
CR0005-1	4/3/20	24	87,434	3,626	2,413	<100	12.34	1.47	5.60	2,496
CR0005-2	4/20/20	41	>312,500	5,957	1,049	145	16.96	1.53	6.11	1,433
CR0005-3	6/22/20	104	20,497	NA	381	NA	NA	NA	NA	471
CR0006-1	4/3/20	20	<100	<100	<100	<100	<0.20	<0.20	0.03	<20
CR0006-2	4/22/20	39	<100	<100	<100	<100	<0.20	<0.20	0.02	<20
CR0006-3	6/25/20	103	<100	NA	<100	NA	NA	NA	NA	<20
CR0007-1	4/6/20	20	9,648	151	556	236	0.83	0.32	1.98	340
CR0007-2	4/24/20	38	23,717	661	378	257	1.95	<0.20	2.30	432
CR0008	4/6/20	29	<100	<100	<100	<100	<0.20	<0.20	0.03	<20
CR0010-1	4/7/20	3	>312,500	22,824	14,636	652	74.13	89.62	6.67	10,283
CR0010-2	4/21/20	39	>312,500	22,517	4,880	177	86.25	37.85	7.28	9,099
CR0010-3	6/22/20	101	76,253	NA	961	NA	NA	NA	NA	1,936
CR0011-1	4/7/20	25	>312,500	7,758	20,308	1,323	21.66	4.58	6.26	2,313
CR0011-2	4/21/20	39	256,413	6,050	4,901	205	17.08	5.49	7.54	4,160
CR0011-3	4/30/20	48	>312,500	2,052	2,996	<100	8.48	1.69	7.21	3,701
CR0012-1	4/7/20	22	15,711	542	658	<100	0.62	<0.20	2.46	226
CR0012-2	4/20/20	35	19,841	796	457	212	1.23	0.21	2.66	253
CR0014-1	4/7/20	14	150,206	6,114	711	108	22.92	0.56	4.36	1,238
CR0014-2	4/21/20	28	>312,500	6,721	369	138	37.12	0.44	3.96	823
CR0016-1	4/8/20	13	<100	<100	<100	<100	<0.20	<0.20	0.01	<20
CR0016-2	4/22/20	27	<100	<100	<100	<100	<0.20	<0.20	0.01	<20
CR0016-3	6/29/20	95	<100	NA	<100	NA	NA	NA	NA	<20
CR0017	4/8/20	21	3,041	118	348	<100	0.27	<0.20	0.80	133
CR0019-1	4/8/20	23	12,457	268	7,247	194	0.81	<0.20	5.47	511
CR0019-2	4/21/20	36	24,908	337	3,164	112	1.44	0.30	5.46	428
CR0019-3	6/17/20	93	3,695	NA	710	NA	NA	NA	NA	105
CR0020-1	4/8/20	19	<100	<100	<100	<100	<0.20	<0.20	0.04	<20
CR0020-2	4/23/20	34	<100	<100	<100	<100	<0.20	<0.20	0.04	<20
CR0021-1	4/9/20	28	>312,500	1,687	1,204	<100	5.60	0.25	5.37	1,582
CR0021-2	4/21/20	40	>312,500	1,916	865	<100	7.80	0.32	6.09	1,263
CR0021-3	6/16/20	96	7,108	NA	257	NA	NA	NA	NA	260
CR0022-1	4/9/20	33	<100	<100	<100	<100	<0.20	<0.20	0.18	<20
CR0022-2	4/22/20	46	<100	<100	<100	<100	<0.20	<0.20	0.16	<20
CR0023-1	4/9/20	27	<100	<100	<100	<100	<0.20	<0.20	0.02	<20
CR0023-2	4/29/20	47	<100	<100	<100	<100	<0.20	<0.20	0.01	<20
CR0024-1	4/10/20	22	<100	<100	<100	<100	<0.20	<0.20	0.03	<20
CR0024-2	4/24/20	36	<100	<100	<100	<100	<0.20	<0.20	0.03	<20
CR0025-1	4/10/20	34	>312,500	2,235	2,035	<100	5.41	1.13	5.29	1,682
CR0025-2	4/23/20	47	>312,500	3,144	813	<100	6.92	1.97	5.40	798
CR0025-3	6/18/20	103	18,497	NA	340	NA	NA	NA	NA	224
CR0026-1	4/13/20	26	4,211	111	7,179	376	0.31	0.52	2.42	587
CR0026-2	4/21/20	34	5,752	190	7,073	366	0.37	0.23	2.81	412
CR0027-1	4/13/20	28	>312,500	4,240	3,973	129	8.76	1.78	6.40	2,284
CR0027-2	6/23/20	99	577,111	NA	9,096	NA	NA	NA	NA	1,959
CR0028	4/14/20	21	<100	<100	<100	<100	<0.20	<0.20	0.08	<20
CR0030-1	4/14/20	24	47,723	1,807	1,251	<100	3.49	2.42	4.26	382
CR0030-2	4/22/20	32	17,404	2,492	2,103	<100	2.99	1.82	3.73	296
CR0032-1	4/15/20	20	<100	<100	<100	<100	<0.20	<0.20	0.01	<20
CR0032-2	4/30/20	35	<100	<100	<100	<100	<0.20	<0.20	0.01	<20
CR0033-1	4/15/20	30	38,557	1,311	3,546	149	2.61	0.22	2.76	282
CR0033-2	4/22/20	37	8,061	1,933	3,596	<100	1.43	<0.20	2.58	452
CR0035-1	4/16/20	29	<100	<100	<100	<100	<0.20	<0.20	0.03	<20
CR0035-2	4/21/20	34	<100	<100	<100	<100	<0.20	<0.20	0.03	<20
CR0035-3	6/18/20	92	<100	NA	<100	NA	NA	NA	NA	<20
CR0037-1	4/16/20	32	15,373	972	3,463	<100	2.73	0.39	1.52	347
CR0037-2	4/23/20	39	20,221	412	2,190	<100	1.87	0.28	1.11	314
CR0037-3	6/25/20	102	4,980	NA	681	NA	NA	NA	NA	110

			Binding antibodies†							Neutralizing antibodies
			Spike (S protein)				RBD		N	
			IgG		IgA		IgG	IgM	IgG	D614G‡
Sample	Date	DFOS	Endpoint titer	EC_{50}	Endpoint titer	EC_{50}	Arbitrary units	Arbitrary units	Index	ID_{50}
CR0038-1	4/16/20	33	24,765	949	677	<100	2.26	<0.20	5.42	50
CR0038-2	4/23/20	40	22,763	420	922	302	1.11	<0.20	5.16	38
CR0038-3	6/17/20	95	6,283	NA	565	NA	NA	NA	NA	125
CR0039-1	4/16/20	30	>312,500	5,706	5,204	145	19.40	1.07	6.55	1,720
CR0039-2	4/23/20	37	>312,500	5,189	7,521	177	12.04	0.79	7.57	633
CR0042-1	4/20/20	41	>312,500	2,653	783	<100	10.24	0.80	7.47	907
CR0042-2	5/8/20	59	94,934	1,412	762	247	7.03	0.64	7.44	747
CR0043-1	4/21/20	37	>312,500	24,185	9,532	367	64.42	3.27	6.65	6,464
CR0043-2	4/29/20	45	>312,500	15,133	15,251	642	40.31	3.55	6.67	7,825
CR0045-1	4/21/20	35	>312,500	21,234	8,748	324	66.45	5.46	7.31	3,283
CR0045-2	4/29/20	43	>312,500	13,698	9,949	249	32.95	5.42	7.00	3,393
CR0046	4/21/20	24	27,663	1,187	3,450	<100	7.16	0.35	7.37	647
CR0048-1	4/15/20	34	36,538	605	1,023	<100	1.42	0.49	3.32	502
CR0048-2	5/28/10	77	10,079	NA	1,003	NA	NA	NA	NA	82
CR0050-1	4/22/20	31	892	<100	<100	<100	<0.20	<0.20	0.02	<20
CR0050-2	4/29/20	38	3,297	<100	<100	<100	<0.20	<0.20	0.02	<20
CR0050-3	6/25/20	95	182	NA	<100	NA	NA	NA	NA	<20
CR0051-1	4/23/20	29	<100	<100	<100	<100	<0.20	<0.20	0.03	<20
CR0051-2	4/30/20	36	<100	<100	<100	<100	<0.20	<0.20	0.03	<20
CR0054-1	4/24/20	43	>312,500	11,252	1,746	112	27.39	0.86	6.63	1,022
CR0054-2	6/26/20	106	33,385	NA	428	NA	NA	NA	NA	597
CR0055-1	4/24/20	33	<100	<100	<100	<100	<0.20	<0.20	0.03	<20
CR0055-2	5/1/20	40	<100	<100	<100	<100	<0.20	<0.20	0.03	<20
CR0057-1	4/27/20	45	>312,500	16,780	5,021	212	32.31	5.23	8.01	4,690
CR0057-2	6/18/20	97	95,234	NA	2,776	NA	NA	NA	NA	1,844
CR0060-1	4/27/20	29	>312,500	21,166	6,772	212	44.54	26.58	7.38	30,472
CR0060-2	6/25/20	88	50,984	NA	608	NA	NA	NA	NA	1,049
CR0061-1	4/27/20	29	>312,500	10,355	15,086	554	21.93	1.07	7.15	1,393
CR0061-2	5/12/20	44	>312,500	10,668	4,778	223	13.36	0.51	6.01	593
CR0061-3	6/19/20	82	61,290	NA	462	NA	NA	NA	NA	695
CR0062-1	4/28/20	36	<100	<100	<100	<100	<0.20	<0.20	0.04	<20
CR0062-2	5/8/20	46	<100	<100	<100	<100	<0.20	<0.20	0.04	<20
CR0064-1	4/28/20	43	3,405	234	671	<100	0.67	0.65	4.53	692
CR0064-2	5/4/20	49	4,541	128	637	107	0.48	0.53	4.22	499
CR0066	4/29/20	39	>312,500	45,653	>312,500	10,578	178.48	10.20	7.53	16,375
CR0067-1	4/29/20	62	13,726	1,265	436	<100	2.58	<0.20	0.72	143
CR0067-2	5/18/20	81	12,486	1,683	319	<100	1.63	0.23	0.37	120
CR0067-3	6/30/20	124	4,153	NA	<100	NA	NA	NA	NA	64
CR0068-1	4/29/20	43	5,663	114	<100	<100	0.45	<0.20	2.26	55
CR0068-2	6/18/20	93	6,306	NA	208	NA	NA	NA	NA	134
CR0069	4/29/20	62	2,393	<100	<100	<100	0.22	<0.20	1.46	44
CR0070-1	4/29/20	42	<100	<100	<100	<100	<0.20	<0.20	0.08	<20
CR0070-2	5/20/20	63	<100	<100	<100	<100	<0.20	<0.20	0.07	<20
CR0071-1	4/30/20	40	>312,500	31,528	18,999	222	115.86	3.18	7.39	3,996
CR0071-2	6/23/20	94	76,966	NA	1,727	NA	NA	NA	NA	1,939
CR0072-1	4/30/20	35	<100	<100	<100	<100	<0.20	<0.20	0.02	<20
CR0072-2	5/19/20	54	<100	<100	<100	<100	<0.20	<0.20	0.01	<20
CR0073-1	4/30/20	49	48,230	1,346	3,726	158	3.98	0.21	6.69	339
CR0073-2	6/12/20	92	10,968	NA	1,697	NA	NA	NA	NA	166
CR0074	5/1/20	43	>312,500	39,059	28,734	499	102.71	2.33	7.48	4,259
CR0078-1	5/1/20	60	210,642	1,910	3,425	144	7.16	0.34	6.23	760
CR0078-2	6/12/20	102	6,306	NA	733	NA	NA	NA	NA	151
CR0079	5/4/20	31	837	<100	361	<100	0.46	<0.20	2.37	69
CR0082-1	5/5/20	40	<100	<100	<100	<100	<0.20	<0.20	0.01	<20
CR0082-2	5/21/20	56	<100	<100	<100	<100	<0.20	<0.20	0.01	<20
CR0083-1	5/5/20	44	<100	<100	<100	<100	<0.20	<0.20	0.04	<20
CR0083-2	5/21/20	60	<100	<100	<100	<100	<0.20	<0.20	0.04	<20
CR0086-1	5/6/20	50	1,569	106	2,352	161	<0.20	<0.20	5.94	92
CR0086-2	6/29/20	108	3,121	NA	1,068	NA	NA	NA	NA	75
CR0087-1	5/7/20	43	<100	<100	<100	<100	<0.20	<0.20	0.23	<20
CR0087-2	5/18/20	54	<100	<100	<100	<100	<0.20	<0.20	0.14	<20
CR0089-1	5/8/20	49	<100	<100	<100	<100	<0.20	<0.20	0.05	<20
CR0089-2	5/27/20	68	<100	NA	<100	NA	NA	NA	NA	<20
CR0090-1	5/11/20	76	<100	<100	<100	<100	<0.20	<0.20	0.01	<20
CR0090-2	6/15/20	111	<100	NA	<100	NA	NA	NA	NA	<20
CR0093-1	5/13/20	63	<100	<100	<100	<100	<0.20	<0.20	0.13	<20
CR0093-2	6/2/20	83	<100	NA	<100	NA	NA	NA	NA	<20
CR0094-1	5/14/20	52	30,955	1,858	2,446	274	10.92	0.65	7.10	1,302
CR0094-2	6/24/20	93	62,626	NA	571	NA	NA	NA	NA	542

			Binding antibodies†							
			Spike (S protein)				RBD		N	Neutralizing antibodies
			IgG		IgA		IgG	IgM	IgG	D614G‡
Sample	Date	DFOS	Endpoint titer	EC_{50}	Endpoint titer	EC_{50}	Arbitrary units	Arbitrary units	Index	ID_{50}
CR0095	5/14/20	54	4,253	1,414	553	<100	1.60	<0.20	1.65	133
CR0098	5/18/20	50	4,570	481	437	<100	1.30	0.48	3.60	158
CR0099	5/18/20	58	<100	<100	<100	<100	<0.20	<0.20	0.02	<20
CR0100	5/19/20	53	>312,500	7,543	844	180	25.46	1.70	4.53	1,011
CR0101	5/19/20	63	<100	<100	<100	<100	<0.20	<0.20	0.02	<20
CR0102	5/20/20	73	>312,500	6,423	1,943	<100	16.99	0.78	6.45	2,080
CR0104	5/22/20	36	>312,500	8,838	33,260	234	22.44	28.18	7.20	19,476
CR0105	5/27/20	72	1,822	<100	<100	<100	0.38	0.56	2.14	99
CR0108	5/28/20	25	<100	<100	<100	<100	<0.20	<0.20	0.01	<20

*SARS-CoV-2, severe acute respiratory syndrome-coronavirus-2; DFOS, days following onset of symptoms (for asymptomatic participants CR0068 and CR0108 d following RT-PCR test were used). NA, not available. Positive values are shown in red.

†Detection of SARS-CoV-2 binding antibodies; Spike, IgG and IgA ELISA reactivities to a pre-fusion stabilized Wuhan-Hu-1 spike protein, with a cutoff at 100 for endpoint and midpoint titers; RBD, IgM and IgG ELISA reactivities to the receptor binding domain of the Wuhan-Hu-1 spike protein with an arbitrary unit cutoff at 0.2; N, detection of IgG responses to the nucleocapsid protein using the Abbott Architect chemiluminescent microparticle immunoassay (CMIA) with an index cutoff at 1.4.

‡D614G, detection of neutralizing antibodies to the D614G variant of the Wuhan-Hu-1 spike using an HIV-1 based pseudovirus assay (see Appendix Methods).

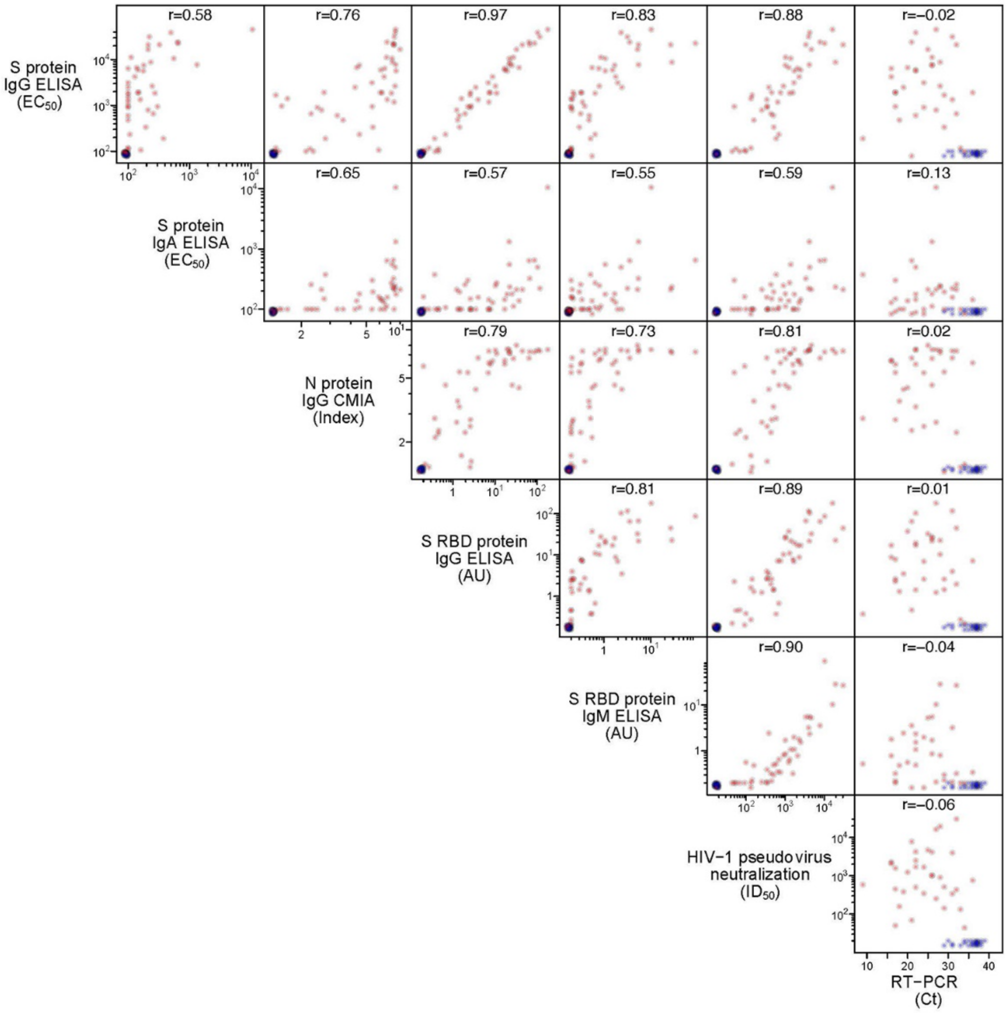

Appendix Figure 1. Comparison of serologic assays detecting SARS-CoV-2 binding and neutralizing antibodies and RT-PCR detecting viral RNA. Each subplot depicts the relationship between measurements from two assays, with half maximal effective concentrations (EC_{50}) plotted for S protein IgG and IgA ELISA titers, signal-to-cutoff index values (Index) plotted for N protein IgG responses (Abbott Architect), arbitrary units (AU) plotted for RBD protein IgG and IgM ELISA titers, half maximal inhibitory doses (ID_{50}) plotted for HIV-1 pseudovirus neutralization titers, and cycle threshold (Ct) values plotted for RT-PCR as listed in Appendix Tables 1 and 2 (x-axes are labeled at the bottom of the column, while y-axes are labeled to the left of the row). Each point represents the maximum titer observed for replicate samples from a given patient and are colored red if any serologic assay for that individual was above the limit of detection (seropositive) and blue if every assay for that individual was below the limit of detection (seronegative). Points below the limit of detection are shown at the limit of detection and offset slightly to aid visualization. The Spearman correlation between the respective assays for seropositive samples is indicated at the top of each subplot (all serologic assay comparisons $p < 0.001$; all RT-PCR vs serologic assays $p > 0.4$).

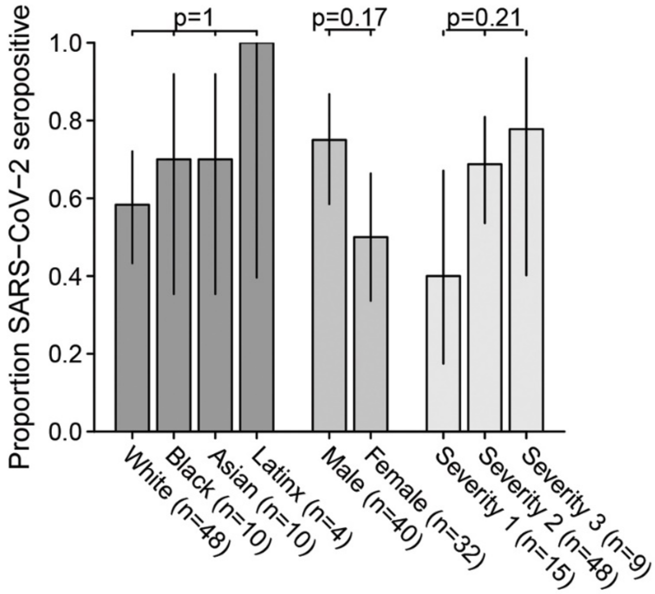

Appendix Figure 2. Relationship of race/ethnicity, sex, and disease severity with SARS-CoV-2 seroconversion. Bars indicate the proportion of serologic responders for the category depicted, with lines indicating the 95% confidence interval for this proportion; p-values are shown for a likelihood ratio test of a logistic regression predicting seropositivity by demographics after Bonferroni correction for multiple comparisons.

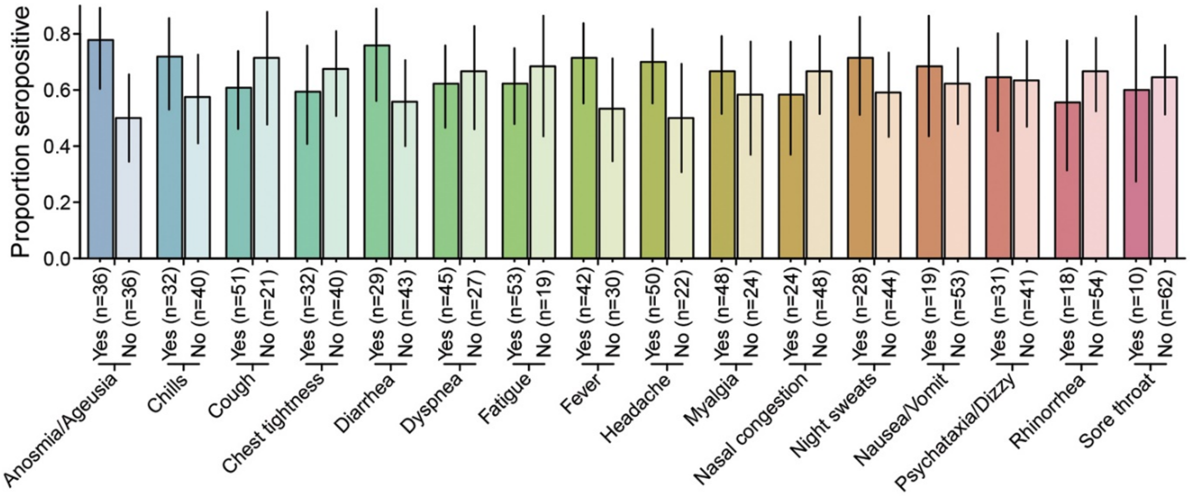

Appendix Figure 3. Relationship of symptoms and SARS-CoV-2 seroconversion. The proportion of serologic responders is compared between persons who reported (light shaded bars) versus did not report (dark shaded bars) one of 16 COVID-19 related symptoms. Vertical lines indicate the 95% confidence interval for each proportion. Likelihood ratio tests of logistic regressions predicting seroconversion by symptom were all nonsignificant after Bonferroni correction for 16 multiple comparisons (all $p > 0.2$). Bars are arbitrarily colored for ease of visualization.

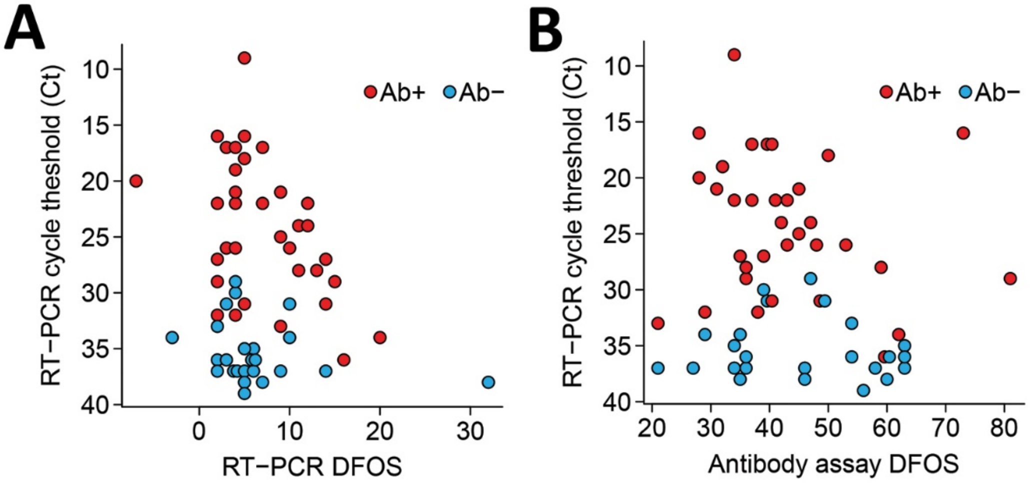

Appendix Figure 4. Comparison of RT-PCR Ct values relative to the time of RT-PCR and serologic testing. Ct values of serologic responders (red) and non-responders (blue) are plotted relative to the time of RT-PCR (A) and serologic (B) testing, measured as days from onset of symptoms (DFOS). For patients with multiple serologic tests, the day of the last sampling is shown. Overlapping points are offset slightly in the x-axis to allow visualization.

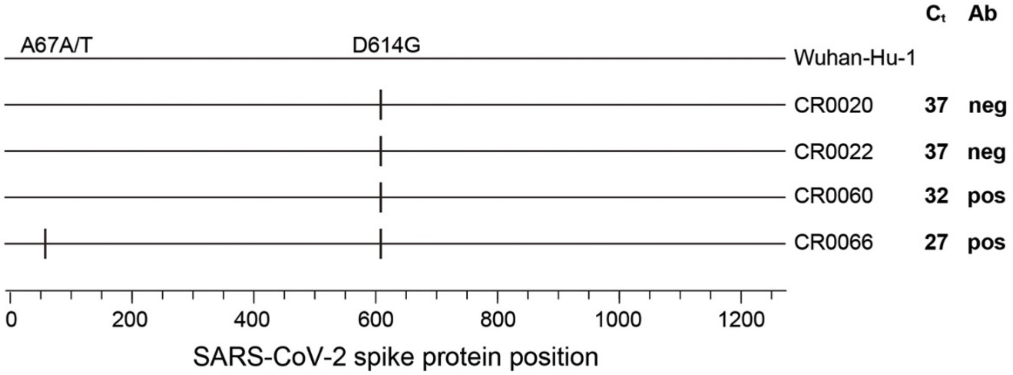

Appendix Figure 5. Amplification of full-length spike sequences from remnant nasal swab materials. A highlighter plot of deduced SARS-CoV-2 spike amino acid sequences is shown for amplicons derived from two serologic responders (CR0060 and CR0066) and two serologic non-responders (CR0020 and CR0022). Amino acid residues that differ from the Wuhan-Hu-1 reference sequence (listed on top) are depicted by vertical marks, with an aspartic acid to glycine substitution at position 614 (D614G) identified in the sequences of all participants and an alanine/threonine mixture at position 67 identified in the sequences of participant CR0066. All genes contain uninterrupted open reading frames. Ct values derived from clinical testing of the same nasal swab materials are indicated.
